# Iron–Sulfur Cluster Biogenesis and Iron Homeostasis in Cyanobacteria

**DOI:** 10.3389/fmicb.2020.00165

**Published:** 2020-02-28

**Authors:** Fudan Gao

**Affiliations:** College of Life Sciences, Shanghai Normal University, Shanghai, China

**Keywords:** Fe–S clusters, SUF mechanism, ISC mechanism, iron homeostasis, cyanobacteria

## Abstract

Iron–sulfur (Fe–S) clusters are ancient and ubiquitous cofactors and are involved in many important biological processes. Unlike the non-photosynthetic bacteria, cyanobacteria have developed the sulfur utilization factor (SUF) mechanism as their main assembly pathway for Fe–S clusters, supplemented by the iron–sulfur cluster and nitrogen-fixing mechanisms. The SUF system consists of cysteine desulfurase SufS, SufE that can enhance SufS activity, SufBC_2_D scaffold complex, carrier protein SufA, and regulatory repressor SufR. The S source for the Fe–S cluster assembly mainly originates from L-cysteine, but the Fe donor remains elusive. This minireview mainly focuses on the biogenesis pathway of the Fe–S clusters in cyanobacteria and its relationship with iron homeostasis. Future challenges of studying Fe–S clusters in cyanobacteria are also discussed.

## Introduction

As cofactors of proteins, iron–sulfur (Fe–S) clusters participate in many important physiological processes, including respiration, photosynthesis, nitrogen fixation, amino acid and purine metabolism, RNA modification, and DNA replication, as well as repair and regulation of gene expression (Beinert et al., 1997; Johnson et al., 2005; Lill, 2009; Balk and Pilon, 2011; Maio and Rouault, 2015). Owing to their photosynthetic autotrophic lifestyle, cyanobacteria are particularly rich in Fe–S clusters. During evolution, cyanobacteria have developed many membrane-embedded photosynthetic protein complexes and electron carriers that contain Fe–S clusters (Table 1). As a consequence, the demand for iron (Fe) in cyanobacteria far exceeds that in other, non-photosynthetic organisms. For example, the Fe quota of oxygenic photosynthetic cyanobacterium *Synechocystis* species strain PCC 6803 (hereafter Synechocystis 6803) cells is one order of magnitude higher than that of non-photosynthetic bacterium *Escherichia coli* (Finney and O’Halloran, 2003; Keren et al., 2004).

**TABLE 1:** Fe–S cluster proteins of photosynthetic complexes in the cyanobacterium *Synechocystis* 6803.

**Complex**	**Open reading frane**	**Protein name**	**Fe–S cluster type**	**References**
PSI	*slr1834/slr1835*	PsaA/PsaB	1 F_x_ ([4Fe–4S])	Jordan et al., 2001
	*ssl0563*	PsaC	1 F_A_([4Fe–4S])	Jordan et al., 2001
	*ssl0563*	PsaC	1 F_B_ ([4Fe–4S])	Jordan et al., 2001
NDH-1	*sll0520*	Ndhl	2 [4Fe–4S]	Laughlin et al., 2019; Schuller et al., 2019
	*slr1280*	NdhK1	1 [4Fe–4S]	Laughlin et al., 2019; Schuller et al., 2019
	*slr8031*	NdhK2	1 [4Fe–4S]	Gao et al., 2020 (in revised)
Cyt *b_6_f*	*sll1316*	PetC	1 Rieske [2Fe–2S]	Kurisu et al., 2003
Ferredoxin	*ssl0020*	Fdx	1 [2Fe–2S]	Cassier-Chauvat and Chauvat, 2014

The Fe–S clusters mainly exist as [2Fe–2S], [4Fe–4S], and [3Fe–4S] types, and their assemblages of Fe ions (+ 2 or + 3 formal oxidation states) and inorganic sulfide (S^2–^) are coordinated to proteins typically by cysteine ligations at each Fe of the Fe–S cluster (Peters and Broderick, 2012) (for reviews, see Beinert, 2000; Lill, 2009; Balk and Pilon, 2011). However, His, Arg, and Glu residues can also be involved in Fe–S cluster coordination (Berkovitch et al., 2004; Meyer, 2008).

The early earth richly contained reducing Fe and S (Wächtershäuser, 1992), and consequently, Fe–S clusters are believed to spontaneously assemble into primitive biological macromolecules by using suitable ligands (Meyer, 2008). The atmosphere started to become oxidized by oxygenic photosynthesis after the proliferation of cyanobacteria between 3.2 and 2.4 billion years ago (Brocks et al., 1999) and severely limited the assembly of Fe–S clusters (Chapman and Schopf, 1983).

Moreover, reactive oxygen species (ROS), as by-product of oxygen metabolism, also damaged Fe–S clusters (Sutton et al., 2004; Wallace et al., 2004). As a consequence, free Fe could produce ROS through a Fenton reaction to damage cells further (Latifi et al., 2009). Under aerobic conditions, a number of dedicated proteins for Fe–S clusters biogenesis are adapted in cyanobacteria. Therefore, an effective balance between Fe acquisition and protection against oxidative stress is critical for cyanobacteria to survive in their habitat. Many researchers have reviewed the assembly of Fe–S clusters in bacteria and plants (Lill, 2009; Balk and Pilon, 2011; Mettert and Kiley, 2015; Lu, 2018). This minireview will focus on the Fe–S cluster biogenesis and its relationship with Fe homeostasis in cyanobacteria. The challenges of studying Fe–S clusters in cyanobacteria are also discussed.

## Fe–S Clusters Biogensis

So far, three major mechanisms have been identified for the assembly of Fe–S clusters, including the nitrogen-fixing (NIF), iron–sulfur cluster (ISC), and S utilization factor (SUF) (Johnson et al., 2005; Lill, 2009). The NIF system is the first discovery of Fe–S cluster biosynthesis pathway in *Azotobacter vinelandii*, and its function is specific to the assembly of Fe–S clusters for the nitrogenase in NIF organisms (Jacobson et al., 1989a, b). Meanwhile, the *isc* gene region was identified in *A. vinelandii* using a biochemical approach, and its products are suggested to participate in Fe–S cluster assembly as housekeeping role and are distributed across almost all domains of life, from some archaea and gram-negative bacteria to yeasts, plants, animals, and humans (Zheng et al., 1998; Lill, 2009; Rouault, 2012). The SUF system is the third discovery of Fe–S cluster biosynthesis pathway (Takahashi and Tokumoto, 2002). Compared with ISC system, SUF system is less widespread and is found only in archaea, most gram-positive bacteria, the chloroplasts of plants, and green algae (Takahashi et al., 1986, 1991; Takahashi and Tokumoto, 2002; Albrecht et al., 2010; Santos et al., 2014; Selbach et al., 2014). In *E. coli*, the SUF system is activated only in response to conditions of oxidative stress or Fe starvation (Outten et al., 2004; Outten, 2015). During evolution, cyanobacteria choose SUF as their major system for Fe–S cluster biosynthesis (Balasubramanian et al., 2006; Ayala-Castro et al., 2008; Outten, 2015), and all core *suf* genes cannot be knocked out completely in the cyanobacterium Synechocystis 6803 (Tirupati et al., 2004; Balasubramanian et al., 2006; Zang et al., 2017). In higher plants, the importance of SUF system has been verified by analyzing its mutants (Xu and Møller, 2006; Hu et al., 2017). The phylogenetic distribution of the SUF system indicates a coevolutionary relationship with photosynthetic energy storing pathways (Zang et al., 2017). This may be a reason why cyanobacteria chose SUF system as their major synthesis pathway for Fe–S clusters.

**FIGURE 1:**
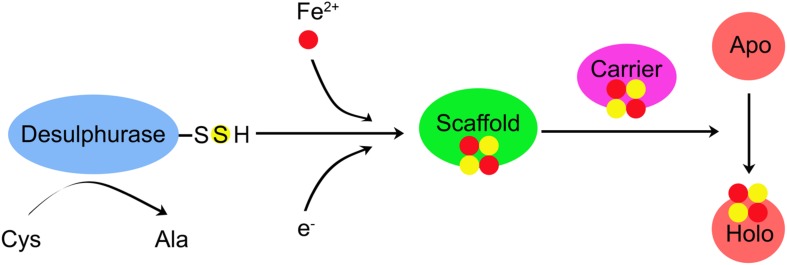
A proposed principle for the Fe–S cluster biogenesis. Three Fe–S cluster systems have been identified in cyanobacteria, including the nitrogen-fixing (NIF), iron–sulfur (Fe–S) cluster (ISC), and S utilization factor (SUF). Three different machines may follow a common biosynthetic rule. The overall biogenesis process can be divided two main steps: (1) *de novo* assembly of Fe–S cluster on the scaffold protein by recruiting Fe and S; (2) transferring Fe–S cluster from the scaffold protein to target apo-proteins (apo-protein) and then are assembled into the polypeptide chain. Cysteine (Cys) is converted to alanine (Ala) by the Cys desulfurase. Electrons are needed for the reduction of S^0^ (Cys) to S^2–^ (Fe–S cluster). The source of Fe is not yet known. *De novo* assembly of Fe–S cluster is performed on the scaffold. The newly assembled Fe–S cluster is transferred to the carrier protein, which delivers the Fe–S cluster to recipient Apo and converts recipient Apo into holo-protein (Holo).

These three different mechanisms follow a common biosynthetic rule. The overall biogenesis process can be divided two main steps: (1) *de novo* assembly of Fe–S cluster on the scaffold protein by recruiting Fe and S and (2) transferring the Fe–S cluster from the scaffold protein to target apo-proteins (Apo) (Figure 1; Lill, 2009; Balk and Pilon, 2011). As shown in Table 2, the main components involved in Fe–S cluster biosynthesis are identified in cyanobacteria using sequence alignment, reverse genetics, physiology, and biochemistry approaches.

**TABLE 2:** Supposed Fe–S cluster biogenesis genes in the cyanobacterium *Synechocystis* 6803.

**Protein name**	**Open reading frame**	**Proposed function**	**Phenotype of mutants**	**References**
**SUF system**				
SufR	*sll0088*	Regulatory repressor	No visible phenotype	Wang et al., 2004; Seki et al., 2006; Shen et al., 2007
SufA	*slr14l7*	Carrier protein, possible iron carrier	No visible phenotype	Morimoto et al., 2002; Wollenberg et al., 2003; Balasubramanian et al., 2006
SufB	*Slr0074*	Fe-S cluster assembly scaffold	Lethal	Balasubramanian et al., 2006; Zang et al., 2017
SufC	*slr0075*	Fe-S cluster assembly component, provide energy	Lethal	Balasubramanian et al., 2006; Zang et al., 2017
SufD	*slr0076*	Fe-S cluster assembly component	Lethal	Balasubramanian et al., 2006; Zang et al., 2017
SufS	*Slr0077*	Cysteine desulphurase sulphur donor	Lethal	Seidler et al., 2001; Tirupati et al., 2004; Balasubramanian et al., 2006; Zang et al., 2017
SufE	*Slr1419*	Enhances SufS activity	Lethal	Balasubramanian et al., 2006; Zang et al., 2017
**ISC system**				
IscR	*Slr0846*	Regulatory represser	Not studied	Uncharacterized
IscSI	*str0387*	Cysteine desulphurase, sulphur donor	No visible phenotype	Seidler et al., 2001; Behshad et al., 2004; Tirupati et al., 2004
lscS2	*sll0704*	Cysteine desulphurase, sulphur donor	No visible phenotype	Seidler et al., 2001; Tirupati et al., 2004
IscA	*slr1565*	Fe-S cluster assembly scaffold, posible iron donor	No visible phenotype	Morimoto et al., 2003
HscA	*sll0170*	Mollecular chaperone	Not studied	Uncharacterized
HscB	*sll0169*	Mollecular chaperone	Not studied	Uncharacterized
Fdx	*Slr0148*	Electron transfer	Not studied	Uncharacterized
**NIF system**				
NifU like	*ssl2667*	Fe-S cluster assembly	Lethal	Nishio and Nakai, 2000; Seidler et al., 2001; Balasubramanian et al., 2006

### SUF Mechanism

In archaea, the components of SUF system are relatively simple, and its minimal functional core consists only of SufBC (Anbar et al., 2007). During evolution from archaea to bacteria, many components of this system are added, including SufA-SufE and SufS (Zheng et al., 2001; Lee et al., 2004; Outten et al., 2004). In oxygenic photosynthetic organisms, cyanobacteria and higher plants retain the components of SUF system in *E*. *coli* and choose this system as their major Fe–S cluster assembly pathways (Outten, 2015). This appears to be an evolutionary choice in response to the rise of oxygen (Boyd et al., 2014).

The SufABCDSE proteins are well characterized in *E*. *coli*. SufA is a scaffold protein that can transfer the [2Fe–2S] cluster into Apo (Ollagnier-de-Choudens et al., 2004; Vinella et al., 2009). SufB forms a stable complex with SufC and SufD with a 1:2:1 stoichiometry, and subsequently, the SufBC_2_D complex functions as a new type of scaffold for the formation of Fe–S clusters (Chahal et al., 2009; Wollers et al., 2010). SufS, a pyridoxal 5′-phosphate-dependent cysteine desulfurase, possesses a low catalytic activity (Mihara et al., 1999, 2000) and can be fully activated upon binding with SufE to form SufSE complex, which can transfer S atoms into SufB (Loiseau et al., 2003; Outten et al., 2003; Layer et al., 2007).

In the cyanobacterial genome, the *sufB*, *sufC*, *sufD*, and *sufS* (*sufBCDS* operon) are arranged with the same transcriptional direction; *sufA* is not included in the *sufBCDS* operon, and *sufR* is located at upstream of *sufB* with an opposite transcriptional direction (Wang et al., 2004; Seki et al., 2006; Shen et al., 2007; Bai et al., 2018). Cyanobacterial SufR can coordinate two [4Fe–4S]^2+, 1+^ clusters and functions as a transcriptional repressor of the *sufBCDS* operon and an autoregulator itself (Shen et al., 2007). The dual functions of SufR depend on the redox state of [4Fe–4S]^2+, 1+^ clusters (Shen et al., 2007). The transcription level of SufR is also regulated by light, oxidative stress, and Fe deficiency (Wang et al., 2004; Seki et al., 2006; Vuorijoki et al., 2017). Specifically, SufR represses the promoter of *sufBCDS* operon (P1, not P2; two promoters P1 and P2 for *sufBCDS* operon) under moderate light conditions, and P1 activation results from the derepression by the high light shift (Seki et al., 2006). Under the conditions of oxidative stress and Fe deficiency, expression levels of the *sufBCDS* genes were elevated in Δ*sufR* (Wang et al., 2004; Vuorijoki et al., 2017). Therefore, *sufR* is also a transcriptional repressor of the *suf* operon under Fe-limiting conditions. Similar to bacterial and plastid SufA, little is known regarding whether cyanobacterial SufA functions as assembly scaffold or carrier with Fe or Fe–S cluster. In the cyanobacterium *Synechocystis* 6803, *in vitro* purified SufA appears to only bind Fe (Morimoto et al., 2002). However, the recombinant protein exists as a dimer that can bind a [2Fe–2S] cluster and then transfer into Apo of [2Fe–2S] and [4Fe–4S] clusters (Wollenberg et al., 2003). As a consequence, deletion of *sufA* exhibited a chlorosis compared with the wild type under Fe-deficient conditions, regardless of a similar growth phenotype under standard growth conditions (Balasubramanian et al., 2006). Similarly, *in vitro* purified plastid SufA can bind a [2Fe–2S] cluster (Abdel-Ghany et al., 2005; Yabe and Nakai, 2006) and transfer the Fe–S cluster into apo-ferredoxin (apo-Fdx) (Abdel-Ghany et al., 2005). However, the phenotype of mutant was the same with wild type even under Fe-deficient conditions in *Arabidopsis* (Yabe and Nakai, 2006). Collectively, it suggested that SufA may be an Fe–S cluster carrier protein and not assembly scaffold in oxygenic photosynthetic organisms. In cyanobacteria, SufBC_2_D is proposed to be a major scaffold complex of Fe–S cluster assembly, although the experimental evidence is absent.

It was previously reported that knockout of each of *sufBCDS* and *sufE* genes was lethal in cyanobacteria (Tirupati et al., 2004; Balasubramanian et al., 2006; Zang et al., 2017) and in higher plants (Xu and Møller, 2006; Hu et al., 2017). This indicates that the SUF system is essential to carry out oxygenic photosynthesis. In cyanobacteria and higher plants, the SUF system was reported to be involved in the biogenesis of the Fe–S clusters for photosystem I (PSI) (Wang et al., 2004; Shen and Golbeck, 2006). In chloroplasts, High Chlorophyll Fluorescence 101 (HCF101) was reported to function as a scaffold protein for assembly of the [4Fe–4S] cluster (Schwenkert et al., 2010). In the Δ*hcf101* mutant, the levels of [4Fe–4S] proteins of PSI were severely reduced in chloroplasts (Lezhneva et al., 2004; Stöckel and Oelmüller, 2004), suggesting that HCF101 may be required for biosynthesis of Fe–S clusters in PSI. Slr0067, a counterpart of HCF101 in Synechocystis 6803 (Lezhneva et al., 2004), and interacts with NdhI, a subunit of NDH-1 complex, as deduced from the results of yeast two-hybrid assay (Dai et al., 2013). NdhI contains two [4Fe–4S] clusters (Laughlin et al., 2019; Schuller et al., 2019). Thus, Slr0067 may be involved in formation of [4Fe–4S] clusters of NDH-1 in cyanobacteria. Furthermore, NDH-1 interacts PSI to form a supercomplex NDH-1-PSI (Peng et al., 2008; Gao et al., 2016), but the interrelationship between Slr0067/HCF101 and the supercomplex needs to be further investigated.

HCF101/Slr0067 is a conserved protein and also exists in non-photosynthetic organisms. The counterpart in *Salmonella enterica* is ApbC that is required for the maturation of the Fe–S clusters proteins in thiamine biosynthetic pathway (Skovran and Downs, 2003; Boyd et al., 2008, 2009). In addition, HCF101/Slr0067 with homology to NBP35 is a P-loop NTPase in cytosolic Fe–S cluster protein assembly (CIA) machinery (Bych et al., 2008; Balk and Schaedler, 2014). NBP35 can interact with Cfd1 (cytosolic Fe–S cluster deficient) to form a heterotetrameric complex as a scaffold in Fe–S cluster protein maturation in yeast and mammals (Netz et al., 2007, 2012; Balk and Schaedler, 2014). However, Cdf1, a counterpart of NBP35, lacks the N-terminal Fe–S cluster-binding domain (Roy et al., 2003; Hausmann et al., 2005) and is also not identified in green lineage (Bych et al., 2008; Kohbushi et al., 2009). Collectively, NBP35 is considered to function as a homodimer and can assemble [2Fe–2S] and [4Fe–4S] clusters on C- and N-terminal domains, respectively, in green lineage (Bych et al., 2008; Kohbushi et al., 2009).

**FIGURE 2:**
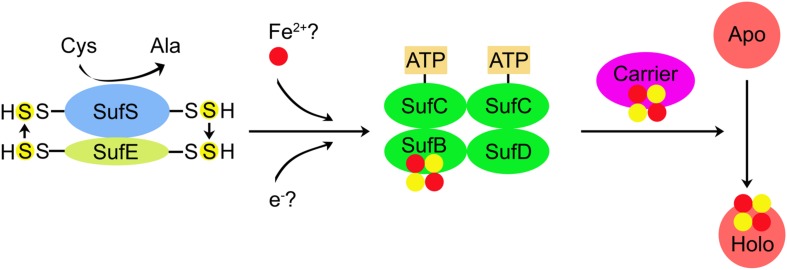
A proposed model for the assembly of Fe–S clusters by SUF system in cyanobacteria. Fe–S cluster biogenesis is initiated by SufS (cysteine desulfurase), which converts cysteine (Cys) to alanine (Ala). Sulfane (S^0^) is transferred from SufS to SufE (sulfur transferase) and then to SufB of SufBC_2_D scaffold complex and bound as a persulfide (S^2–^). Putative Fe and electron (for reduction S^0^ to S^2–^) donors are still unknown. SufC has an ATPase activity, thus coupling ATP hydrolysis with the formation of Fe–S clusters. Subsequently, the newly assembled Fe–S cluster is transferred to the carrier protein, which delivers the Fe–S cluster to apo-protein (Apo) and further converts Apo to holo-protein (Holo). SufA and Slr0067 (*Synechocystis* 6803) may function as the carrier proteins.

Based on the above analyses, a model of cyanobacterial SUF system for Fe–S cluster synthesis is schematically represented in Figure 2. Fe–S cluster biogenesis is initiated by SufS, which converts L-cysteine (Cys) to L-alanine (Ala). Sulfane (S^0^) is transferred from SufS to SufE (S transferase) and then to SufB of SufBC_2_D scaffold complex and bound as a persulfide (S^2–^). Putative Fe and electron (for reduction of S^0^ to S^2–^) donors are still unknown. SufC has an ATPase activity, thus coupling ATP hydrolysis with the formation of Fe–S clusters. Subsequently, the newly assembled Fe–S cluster is transferred to the carrier protein, which delivers the Fe–S cluster to Apo to form holo-protein (Holo).

### ISC Mechanism

Cyanobacterial genome contains almost all homologs of ISC system from *E*. *coli* (Table 2), although this system is less important in cyanobacteria. The ISC assembly system encoded by *iscR*-*iscSUA*-*hscBA*-*fdx* has been well studied in *E. coli*. Among them, IscR suppresses the expression of gene cluster *isc* (Fleischhacker et al., 2012) as a global regulator for Fe–S cluster biogenesis (Schwartz et al., 2001). IscS is pyridoxal 5′-phosphate–dependent cysteine desulfurase (Flint, 1996), and it is also a major cysteine desulfurase that can catalyze the reaction of L-cysteine to L-alanine and lead to release the S element required for Fe–S cluster formation (Schwartz et al., 2000). Two cysteine desulfurases (IscS1 and IscS2) were previously identified in cyanobacteria, but their absence did not affect the growth of cells under normal growth conditions (Seidler et al., 2001; Behshad et al., 2004; Tirupati et al., 2004). Although IscS1 and IscS2 were absent, SufS may supply S for the ISC system. SufS is essential for the growth and thus plays a dominant role in cysteine desulfurization for Fe–S cluster biogenesis in cyanobacteria. In contrast, the function of IscS1 and IscS2 on cysteine desulfurization is relatively minor. Consequently, deletion of *iscS1* and *iscS2* did not affect the growth of cyanobacterial cells. Two heat shock cognate proteins, HscB and HscA, specifically interact with IscU (Silberg et al., 2004; Tapley and Vickery, 2004) and promote an ATP-dependent reaction that the assembled Fe–S clusters are transferred from IscU into Apo (Chandramouli and Johnson, 2006; Bonomi et al., 2008; Alderson et al., 2014). It is worthy of note that a typical IscU is missing in some non–nitrogen-fixation cyanobacteria, for example, Synechocystis 6803 (Kaneko et al., 1996; Seidler et al., 2001). This may be that IscU mainly functions for the assembly of Fe–S clusters proteins related to nitrogenase in NIF organisms.

IscA is a scaffold for Fe–S cluster assembly (Ding and Clark, 2004) that can transfer [2Fe–2S] cluster into Apo (Ollagnier-de-Choudens et al., 2004). Ferredoxin may provide electrons for the Fe–S cluster assembly (Chandramouli et al., 2007; Shi et al., 2012). In *Synechocystis* 6803, IscA can also bind a [2Fe–2S] cluster, but the presence of IaiH (IscA-interacting Heat-repeats–containing protein) is required for their stable binding (Morimoto et al., 2003). Only three cysteine residues are conserved in IscA (Morimoto et al., 2002), and IaiH may be required to provide another cysteine to further stabilize the [2Fe–2S] cluster. Although IscA is able to bind [2Fe–2S] cluster *in vitro* in the absence of IaiH (Morimoto et al., 2002, 2003), it was shown that nearly all cellular IscA and IaiH exist as a complex (Morimoto et al., 2003). This suggests that IscA interacts with IaiH to form a complex that may perform physiological functions *in vivo*. The functions of other members of cyanobacterial ISC system need to be further investigated in the future.

### NIF Mechanism

In the cyanobacterial NIF system, only one scaffold protein NfuA is involved in Fe–S cluster assembly. Nfus are U-type proteins and contain a typical Nfu domain that shares a high sequence identity with the C-terminal domain of NifU (Angelini et al., 2008). The binding forms of NfuA with Fe–S cluster in cyanobacteria are different. In the cyanobacterium *Synechocystis* 6803, *in vitro* purified NfuA can transfer a labile [2Fe–2S] cluster into apo-Fdx (Nishio and Nakai, 2000). By contrast, in the cyanobacterium *Synechococcus* species PCC 7002, NufA can transfer the [4Fe–4S] cluster into PsaC, a subunit of PSI complex, via their interaction (Jin et al., 2008). Furthermore, complete segregation of Δ*nfuA* mutant was not obtained, indicating that NfuA is indispensable for cell growth and supporting that NfuA functions as the scaffold protein in the NIF system (Seidler et al., 2001; Balasubramanian et al., 2006).

In order to perform the Fe–S cluster assembly of nitrogenase in *A. vinelandii*, a series of genes (*nifUSVWZM*) are necessary. They gradually lose the function of biological nitrogen fixation in cyanobacteria, possibly because of the purpose of carrying out photosynthesis. As a consequence, their encoding products retain only one scaffold protein to involve in Fe–S cluster assembly. Higher plants have completely lost the NIF mechanism during evolution.

## Iron Homeostasis

Iron and S meet at the scaffold protein, leading to the biosynthesis of Fe–S clusters. Release of an excessive free Fe damages cyanobacterial cells, regardless of the fact that Fe is important for Fe–S cluster synthesis. As a consequence, it is very important to maintain Fe homeostasis in cyanobacterial cells. It has been proposed that Fe donor or carrier and Fe storage proteins play an important role in Fe homeostasis.

### Iron Donor

It is well known that S for the Fe–S cluster assembly comes from L-cysteine catalyzed by desulfurase SufS or IscS. However, Fe donor remains elusive. Frataxin is an important mitochondrial protein and its decrease causes Friedreich’s ataxia (FRDA), a lethal neurodegenerative disease (Campuzano et al., 1996). This protein has been proposed as a possible Fe donor for the Fe–S cluster biogenesis (Yoon and Cowan, 2003; Layer et al., 2006). Frataxin was further found to interact with the S donor IscS and the scaffold protein IscU for Fe–S cluster biogenesis (Layer et al., 2006; Adinolfi et al., 2009; Shi et al., 2010). BLAST searches unveiled that frataxin is highly conserved from bacteria to human (Babcock et al., 1997) but is absent in the genome of cyanobacteria^1^. Based on previous studies, we speculate that there are several reasons for the absence of frataxin in cyanobacteria: (1) regardless of a phylogenetic co-occurrence of frataxin with proteins of the Isc operon in cells (Huynen et al., 2001), ISC system is not a main Fe–S assembly machine in cyanobacteria; (2) frataxin and its homologs have a weak Fe-binding activity (Ding et al., 2007; Lu et al., 2010; Stemmler et al., 2010), inconsistent with the high-Fe demand in cyanobacteria. To cope with the high-Fe demand, it is logical to hypothesize that cyanobacteria lose frataxin with low-Fe affinity. During evolution, it appears plausible that cyanobacteria might have chosen a protein with high-Fe affinity as their Fe donor, although we do not know who this protein is.

Alternative Fe donor proteins are suggested to be IscA and SufA because they have a high affinity for Fe-binding activity in *E. coli* and cyanobacteria (Wollenberg et al., 2003; Ding et al., 2004; Lu et al., 2008; Landry et al., 2013). Unfortunately, these studies are carried out *in vitro*, and Fe donors proposed have not been shown to interact with cysteine desulfurases or scaffold proteins (Py and Barras, 2010).

Moreover, a phenotype analysis under standard growth conditions has failed to provide any strong evidence that supports a role for IscA/SufA in cellular Fe homeostasis (Seidler et al., 2001; Djaman et al., 2004; Balasubramanian et al., 2006). Therefore, IscA/SufA may only be used for transferring Fe or Fe–S cluster into Apo as carrier protein. However, there is a notable and interesting question that there are subtle regulatory mechanism defects in IscA/SufA. Absence of IscA will result in mistakenly sensing Fe limitation in cyanobacterial cells as deduced from the increased Fe stress-induced protein A (IsiA) protein, regardless of the fact that cells are under the Fe-sufficient conditions (Balasubramanian et al., 2006). IsiA is chlorophyll *a*-binding protein that forms around PSI under Fe limitation and thus is usually selected as a marker for Fe deficiency in cyanobacteria (Melkozernov et al., 2003; Ryan-Keogh et al., 2012). Nevertheless, the inappropriate Fe limitation response in Δ*iscA* is ameliorated by additionally inactivating the *suf* gene (Balasubramanian et al., 2006). Thus, IscA plays an important role in sensing to Fe levels in cyanobacterial cells.

### Iron Storage Protein

Iron storage proteins are considered to be important ways for regulating Fe homeostasis in cyanobacteria. Two types of Fe storage proteins are present in cyanobacteria: bacterioferritin (BFR) and DNA-binding proteins from starved cells (DPS) (Keren et al., 2004; Castruita et al., 2006; Shcolnick et al., 2007). These storage proteins are involved in the storage, release, and transfer of Fe. As a consequence, they play an important role in Fe homeostasis.

In cyanobacteria, multiple *bfr* genes are present in genome (Keren et al., 2004). Bfr proteins have heme or di-Fe binding site in response to different physiological functions. In *E. coli*, it has been reported that hemeless Bfr accumulates four times more Fe than a Bfr that binds heme, *in vitro* (Andrews et al., 1995). This suggests that while the di-Fe center is needed for Fe acquisition, the heme may be needed for Fe extraction from the Bfr structure. Bfrs store Fe in a cavity at the center of their 24-mer ultrastructure. Iron enters the Bfr complex as Fe^2+^ and is oxidized on its way to the central cavity (Carrondo, 2003; Lewin et al., 2005). In cyanobacterium *Synechocystis* 6803, there are two *bfr* genes, *bfrA* and *bfrB*. Targeted mutagenesis of each of them resulted in poor growth under Fe-deprived conditions (Keren et al., 2004), however, inactivation of both genes did not cause a more severe phenotype (Keren et al., 2004). This result suggests the possible presence of a heteromultimeric structure of cyanobacterial BFR, in which one subunit ligates a di-Fe center, whereas the other accommodates heme binding.

DNA-binding proteins from starved cells proteins are a subgroup of the ferritin family that lack the fifth helix found in other ferritins (Andrews et al., 2003). During evolution, DPS divided into different functions. It functions as Fe storage proteins, DNA-binding proteins protecting against oxidative stress, cold shock proteins, neutrophile activators, and pili components (Andrews et al., 2003). In cyanobacterium *Synechococcus* species PCC 7942, DpsA binds a heme (Peña and Bullerjahn, 1995), and inactivation of DpsA results in slow growth rates on the Fe-depleted media (Sen et al., 2000). However, a Dps family protein MrgA in cyanobacterium Synechocystis 6803 cells appears to have a specific role in intracellular Fe trafficking, rather than in Fe storage (Shcolnick et al., 2007). MrgA can catalyze similar reactions as BFR, oxidizing Fe^2+^ to Fe^3+^ using hydrogen peroxide (H_2_O_2_) (Lewin et al., 2005). However, MrgA may be located downstream of BFR and may not affect the total Fe storage. It coordinates the dynamic balance of Fe *in vivo* mainly through BFR (Li et al., 2004; Shcolnick et al., 2007, 2009). Therefore, Fe storage proteins are an important strategy for cyanobacteria to regulate Fe balance and protect cells.

## Differentiation of Fe–S Cluster Pathways Between Cyanobacteria and Bacteria

Although cyanobacteria inherit the biosynthetic pathways of Fe–S clusters, changes have taken place in the process of using these pathways to synthesize Fe–S clusters. Cyanobacteria choose SUF mechanism, which has higher tolerance to oxidative stress in bacteria as the main Fe–S cluster assembly pathway, supplemented by ISC and NIF mechanisms.

In bacteria, ISC is the housekeeping Fe–S cluster assembly system (Lill, 2009; Ding, 2016), whereas SUF is induced when bacteria encounter Fe-limited or oxidative stress (Outten et al., 2004; Outten, 2015). However, cyanobacteria adopt a different Fe–S cluster assembly strategy from bacteria. Sulfur utilization factor is a dominating Fe–S cluster assembly mechanism, whereas ISC mechanism is auxiliary in cyanobacteria. It is possible that the Fe–S cluster synthesis system in cyanobacteria is distinct from other prokaryotes for several reasons: (1) cyanobacteria are prokaryotes with photosynthetic characteristics, in which abundant Fe–S cluster proteins participate in photosynthetic electron transport in thylakoid membrane (Table 1). For example, consistent with *Arabidopsis thaliana*, SufA in the cyanobacterium Synechocystis 6803 contains five Cys residues, however, IscA contains only three Cys residues in non-photosynthetic organisms (Balasubramanian et al., 2006). Because SUF system may be involved in Fe–S cluster assembly of PSI (Yu et al., 2003; Wang et al., 2004), the components specific to the assembly of the Fe–S clusters in photosynthetic complexes were formed during evolution. (2) The reduced bioavailability of Fe and S by oxygenic photosynthesis drives the production of additional components of SUF system in response to the oxidative stress. Under conditions of anaerobic or very low concentration of oxygen, the core SufBC scaffold complex is sufficient to assemble Fe–S clusters protein because of presence of the majority of soluble Fe^2+^ and S^2–^ (Boyd et al., 2014). With the increase in oxygen levels, SufD, SufS, and SufE are added into the SUF system in order to adapt an environment of decreased bioavailability of Fe and S (Boyd et al., 2014). Undoubtedly, cyanobacteria choose the SUF system as a dominant Fe–S cluster biosynthetic mechanism. (3) Reactive oxygen species produced by oxygenic metabolism from photosynthetic electron transport and other oxygenic metabolism pathways will damage the Fe–S clusters in proteins. Excessive electron accumulation in photosystem II (PSII) and PSI, especially under high light stress conditions, will combine with oxygen to produce ROS directly damaging Fe–S clusters. Sulfur utilization factor system is activated by high light and promotes Fe–S cluster biogenesis to compensate for the high light stress (Seki et al., 2006). Furthermore, free Fe could produce more deleterious ROS through a Fenton reaction to damage cyanobacterial cells. Collectively, in order to cope with the side effects of photosynthesis, cyanobacteria primarily select the SUF system to assemble Fe–S clusters and optimize this system to adapt to their inhabit environment.

## Perspectives

Fe–S cluster proteins are essential for many biological processes. During evolution, three assembly pathways for Fe–S clusters, SUF, ISC, and NIF, are formed in cyanobacteria. Over several decades, despite many progresses in biosynthesis of Fe–S clusters, thorough basis structure, detailed biochemical characteristics, and functional molecular mechanism are yet unknown. Some key components specific to the Fe and electron donors of SUF machinery for Fe–S cluster biogenesis need to be further characterized. Additionally, cyanobacteria inherited an SUF system from bacteria, but this system in cyanobacteria has a higher tolerance to oxidative stress in comparison with that in bacteria because of high oxidative stress raised by oxygenic photosynthesis. However, the functional mechanism is not yet uncovered. It has been proposed that the SUF system may be associated with the biosynthesis of Fe–S clusters in photosynthetic membrane protein complexes, including PSI and NDH-1 (Lezhneva et al., 2004; Stöckel and Oelmüller, 2004; Dai et al., 2013). With the exception of Slr0067, however, no other Fe–S assembly proteins of the SUF system have been identified to interact with the photosynthetic membrane protein complexes in cyanobacteria.

The functional roles of many components of Fe–S cluster assembly systems identified in cyanobacteria were proposed based on their counterparts in bacteria and higher plants. To unravel the specific roles of these components and the regulatory network of Fe–S cluster assembly and transfer pathways, further studies are required in cyanobacteria in the future.

## Author Contributions

FG wrote the manuscript.

## Conflict of Interest

The author declares that the research was conducted in the absence of any commercial or financial relationships that could be construed as a potential conflict of interest.

## Footnotes

1 http://genome.annotation.jp/cyanobase
